# Identification of Cuproptosis‐Related Patterns Predict Prognosis and Immunotherapy Response in Hepatocellular Carcinoma

**DOI:** 10.1111/jcmm.70224

**Published:** 2024-12-11

**Authors:** Kai Guo, Tianbing Wang, Jimin Yin, Shoushan Yang, Haodong Cui, Zichuan Cao, Qiang Zhao, Gongbo Xie, Jian Lu, Guosheng Gu, Wenyong Wu

**Affiliations:** ^1^ Department of General Surgery Anhui No.2 Provincial People's Hospital Hefei China; ^2^ Anhui No.2 Provincial People's Hospital Clinical College of Anhui Medical University Hefei China; ^3^ Anhui No.2 Provincial People's Hospital Hefei China; ^4^ The Fifth Clinical Medical College of Anhui Medical University Hefei China; ^5^ Department of General Surgery The Fourth People's Hospital of Lu'an China

**Keywords:** cuproptosis, hepatocellular carcinoma, immune infiltration, prognostic signature

## Abstract

A novel copper‐dependent mode of death, cuproptosis, has been newly identified. This study developed a cuproptosis score (CS) based on the cuproptosis model to analyse the association of CS with prognosis, immune cell infiltration, drug sensitivity and immunotherapy response in hepatocellular carcinoma (HCC) patients. A typing model of cuproptosis was constructed based on the expression of 19 cuproptosis‐related genes (CRGs). A total of 485 samples were divided into high scoring group (HSG) and low scoring group (LSG) according to CS, and the drug sensitivity and responsiveness to immunotherapy were evaluated by combining the immunophenotype score (IPS), oncoPredict, the tumour immune dysfunction and rejection (TIDE). The use of weighted gene coexpression network analysis (WGCNA) identified key prognostic genes for cuproptosis. Western blotting was used to detect the expression level of the key gene. The CRG key gene glutaminase (GLS) is highly expressed in HCC, and patients with high expression of GLS have a poorer prognosis. Furthermore, cell function experiments, such as proliferation, migration and invasion assays, confirmed that GLS knockdown significantly changed the incidence and progression of HCC. This study suggests that new biological markers associated with cuproptosis can be used in the clinical diagnosis of HCC patients to predict prognosis and therapeutic targets.

## 1 Introduction

Hepatocellular carcinoma (HCC) is one of the common malignancies in humans and its incidence is increasing year by year. According to the global cancer statistics in 2020, HCC has become the third leading cause of cancer‐related death [1]. Among the causative factors of HCC, HBV, HCV, aflatoxin exposure and nonalcoholic steatohepatitis are the most dangerous [2]. The clinical management of HCC has been a problem that has plagued mankind. Despite the efforts made in the treatment of HCC, most patients still have a high rate of recurrence and metastasis [3]. The use of immune checkpoint inhibitor (ICI) therapy targeting PD‐1/PD‐L1 and CTLA‐4 has progressed in a variety of cancers [4]. Nivolumab (PD1 inhibitor) prolongs patient survival to some extent, but is responsive in less than 20% of HCC patients [5]. Thus, understanding the combination of patient stratification and biomarkers to improve HCC treatment outcomes is urgently needed to be addressed.

There are two types of cell death: accidental cell death (ACD) and regulatory cell death (RCD), which is also known as programmed cell death (PCD) [6]. Apoptosis is involved in a variety of pathophysiological processes, including tumour progression and homeostasis [7]. Many emerging RCD modalities have attracted great attention in recent years, including: apoptosis, necroptosis, cytokinesis, iron death, autophagy‐dependent cell death, immunogenic cell death, alkaline prolapse, lysosome‐dependent cell death, entotic cell death, netotic cell death, and so on [8, 9]. Induction of apoptosis in tumour cells has been the key to target cell survival or proliferation pathways and is an important modality for tumour microenvironment therapy [10]. The inhibitory effect of necroptotic apoptotic factor receptor–interacting protein kinase 3 (RIPK3) in colorectal cancer has been reported, and decreased RIPK3 expression significantly reduces overall survival (OS) [11]. Ferroptosis has the ability to activate immune cells in tumours by delivering chemotactic signals, and iron death inducers play a role in suppressing tumour immunotherapy [12]. Recently, a study by Tsvetkov et al. [13] identified a new mode of copper‐induced cell death: cuproptosis. Cuproptosis depends on the aggregation of intracellular copper content, which is a unique way different from other known cell death pathways. Copper sag relies on mitochondrial respiration rather than ATP, lipoylated components from the tricarboxylic acid (TCA) cycle bind to copper and the loss of lipoylated components with iron–sulphur cluster proteins leads to activation of proteotoxicity, resulting in cell death [13]. The involvement of copper in the progression, development and metastasis of various tumours has been previously described [14, 15, 16, 17]. The mechanism of copper‐dependent death mode in tumours has not been well studied, and there is no analysis on copper death‐related gene patterns in HCC prognosis and prediction of immunotherapy response.

In this study, we combined cuproptosis‐related genes with HCC samples from TCGA and GEO databases to identify the subtype pattern of cuproptosis. This study provides new ideas and methods for clinical immunotherapy planning and patient management.

## 2 Materials and Methods

### 2.1 Data Collection and Processing

HCC datasets were from TCGA (http://portal.gdc.cancer.gov/repository) and GSE76427 dataset and GSE27150 dataset in GEO database (http://www.ncbi.nlm.nih.gov/geo/). We also obtained expression profiles and clinicopathological information from GEO datasets. Somatic mutation data for TCGA‐LIHC samples were obtained from UCSC Xena (http://xena.ucsc.edu/) website. The ‘SVA’ R package was used to eliminate the presence of batch and latent variables in TCGA and GEO data normalization [18]. IMvigor210 cohort data were obtained from (http://research‐pub.gene.com/IMvigor210CoreBiologies/) [19]. Perl software (http://www.perl.org/) and R (Version 4.2.0) were used to normalise the above data.

### 2.2 Consensus Clustering Analysis

The ‘limma’ R package was used to analyse the expression of the 19 CRGs. Consistency analysis was usually used to cluster and type the data with the ‘ConsensusClusterPlus’ R package. The ‘survival’ and ‘survminer’ packages were used to analyse the significance of survival between clusters. The clustering heatmap was drawn with the ‘pheatmap’ package.

### 2.3 Functional Enrichment Analysis

To investigate the biological functions between cuproptosis clusters in HCC, gene set variation analysis (GSVA) was performed [20]. The ‘c2.cp.kegg.v7.5.1.symbols.gmt’ annotated gene sets were downloaded from the MSigDB database and the ‘GSVA’ package was used to identify gene set enrichment pathways. The criterion for statistical significance between clusters was set at an adjusted *p* < 0.05.

### 2.4 Evaluation of Immune Cell Infiltration

To evaluate the differential ICI in clusters, we used single‐sample gene set enrichment analysis (ssGSEA) [21]. ‘GSEABase’ and ‘GSVA’ were used to achieve immune evaluation.

### 2.5 Identification and Functional Annotation of Differentially Expressed Genes Between Cuproptosis Clusters

Identification of differentially expressed genes (DEGs) between different cuproptosis clusters was achieved by the ‘Limma’ package. We identified DEGs between clusters based on corrected *p* < 0.001 and |log2FC| > 1. To investigate the possible functional pathways of DEGs in each Cuproptosis cluster, the ‘clusterProfiler’ package was used to analyse the functional enrichment of intersecting genes GO and KEGG [22].

### 2.6 Establishment of Cuproptosis Score

To quantitatively assess the relationship between cuproptosis pattern and HCC, the concept of CS was introduced. On the basis of intersection DEGs, univariate Cox analysis was used to obtain genes significantly related to prognosis and extract the expression amount. Then the CS score was calculated based on the expression of these genes, and the PCA principal component analysis formula was quoted: ΣPCA1i+ΣPCA2i (*i* is the expression amount). The optimal cut‐off value was estimated by combining CS and survival data, and the cut‐off value was used to classify the sample CS into HSG and LSG.

### 2.7 Multiple Treatment Sensitivity Analysis of HSG and LSG Groups

Tumour mutation burden (TMB) generates new immunogenicity and is thought to predict immune checkpoint blockade response [23]. Mutation maftools for HSG and LSG were also plotted to visualise the frequency and type of mutated genes.

The expression data and sensitivity data of targeted drugs are from genetics of drug sensitivity in cancer (GDSC) (https://www.cancerrxgene.org/) obtained, including inhibitors of mTOR, Wnt and other pathways. The drug sensitivity between groups is calculated by ‘oncopredict’ and ‘parallel’ packages [24]. We examined the relationship between the expression of immune checkpoint (ICK) and CS score. IPS for ICK inhibitors by TCIA Database (https://tcia.at/) obtained.

Immune checkpoint inhibition therapy is a current research priority for therapeutic treatment, with PD1, PD‐L1 and CTLA4 the most targeted targets, with only one‐third of treatment responses in patients [25]. The TIDE website (http://tide.dfci.harvard.edu) can predict ICB responses using untreated tumour transcriptome data.

### 2.8 Identification of Key Genes for Cuproptosis by WGCNA

We used weighted gene coexpression network analysis (WGCNA) to identify cuproptosis key genes from prognosis‐related DEGs and CS traits. The cuproptosis key genes were obtained by taking the intersection of the obtained significant module genes with the 19 CRGs.

Validation of expression and prognosis of key genes at the RNA level involves data from TCGA. The protein‐level validation of CRG key genes was performed using the CPTAC database (https://proteomics.cancer.gov/programs/cptac) [26]. Protein expression data and clinical information were obtained from this database, and the gene expression and prognostic significance were analysed.

### 2.9 Human Cancer Specimen Collection and Cell Culture

The paired tumour and adjacent nontumour tissues utilised in this investigation were obtained with the consent of HCC patients at Anhui No. 2 Provincial People's Hospital (Hefei, China) undergoing partial hepatectomy. All patients did not receive any preoperative treatment. Each patient gave their approval to the study. The Biomedical Ethics Committee of Anhui Medical University examined and approved this work.

Six human HCC cell lines (MHCC‐LM3, SMMC‐7721, PLC/PRF/5, HepG2, MHCC97‐H and Hep3B) were purchased from Hunan Fenghui Biotechnology (Changsha, China). STR analysis has confirmed the validity of every cell line used in this investigation (Servicebio, Wuhan, China) and mycoplasma contamination tests have been performed. HCC cells were cultured with Dulbecco's modified Eagle's medium (DMEM) (Cibco, Massachusetts, USA) added the 10% foetal bovine serum (FBS) (Gibco, USA) under the conditions of 37°C and 5% CO_2_ atmospheric composition.

### 2.10 Plasmids, Cell Transfection and Lentivirus

Genechem, located in Shanghai, China, developed and synthesised the sh‐INHBB and the negative control shNC. Lipofectamine 3000 (Invitrogen, L3000001, USA) was used as the transfection reagent in this investigation. In order to transfect cells, when the growth density of target cells reaches 70%–80%, the transfection reagent and plasmid are mixed into the cells according to the ratio of 1:1, and then the cells are cultured for 48 h. Sequences of GLS sh1 and GLS sh2 were 5‐GCACAGACATGGTTGGTATAT‐3 and 5‐CCATAAGAATCTTGATGGATT‐3 respectively. The infected cells were selected with puromycin treatment.

### 2.11 Western Blotting

Hepatocellular carcinoma tissues and adjacent tissues were treated with RIPA lysate (Servicebio, Wuhan, China) to extract proteins. The polyacrylamide 8% SDS‐PAGE gel electrophoresis was prepared. After electrophoresis, membrane transfer, electroporation, staining and sealing, diluted primary antibody was added to the incubation box: β‐action (20536‐1‐AP) and GLS antibody (29519‐1‐AP) were incubated at 4°C overnight. Secondary protest was added after incubation, and ECL luminous solution (Servicebio, Wuhan, China) was used to display the strip.

### 2.12 Functional Experiments on Proliferation, Invasion and Migration In Vitro

CCK‐8 was used to measure the in vitro proliferation of HCC cells (Servicebio, G4103, China). Ninety‐six‐well plates were seeded with 1 × 10 [3]/well cells, and this procedure was repeated six times for the control and experimental groups. Following a 1:10 addition of CCK‐8 to the corresponding cell solution, the cells were incubated for 2 h. A microplate reader (BioTek, USA) was used to measure the absorbance at 450 nm during the designated growth periods (24, 48, 72 and 96 h).

To conduct an invasion and migration experiment, HCC cells were seeded into transwell chambers that were either uncoated or covered with Matrigel. Eight hundred microlitres of DMEM containing 20% FBS was then added to the lower chambers. The transwell cells were incubated for 24 h, after which they were fixed with methanol, stained for 10 min with crystal violet and photographed.

In a six‐well plate, HCC cells were injected for the wound healing experiment. Following the microscopically detected entry of the cells into the logarithmic growth phase, a 10‐μL pipette tip scratch was produced in the corresponding holes. The cells were then gently rinsed twice with PBS to eliminate floating and broken cells, and the appropriate media were added before the cells were cultured for 48 h. Pictures were taken at 0 and 48 h.

### 2.13 Statistical Analyses

The statistical analyses involved in the study were performed in R (version 4.2.0). Wilcox test was used for the analysis of differences without using groups. the Kaplan–Meier method was used to plot survival curves by log‐rank test for differences between groups. Correlation analysis was calculated using Spearman's coefficient. The ‘RCircos’ package was used to plot circles of human chromosomal mutation loci. For the above analysis, the reference standard for statistical significance was *p* < 0.05.

## 3 Results

### 3.1 Genetic Profile of Cuproptosis‐Related Genes in HCC

**FIGURE 1:**
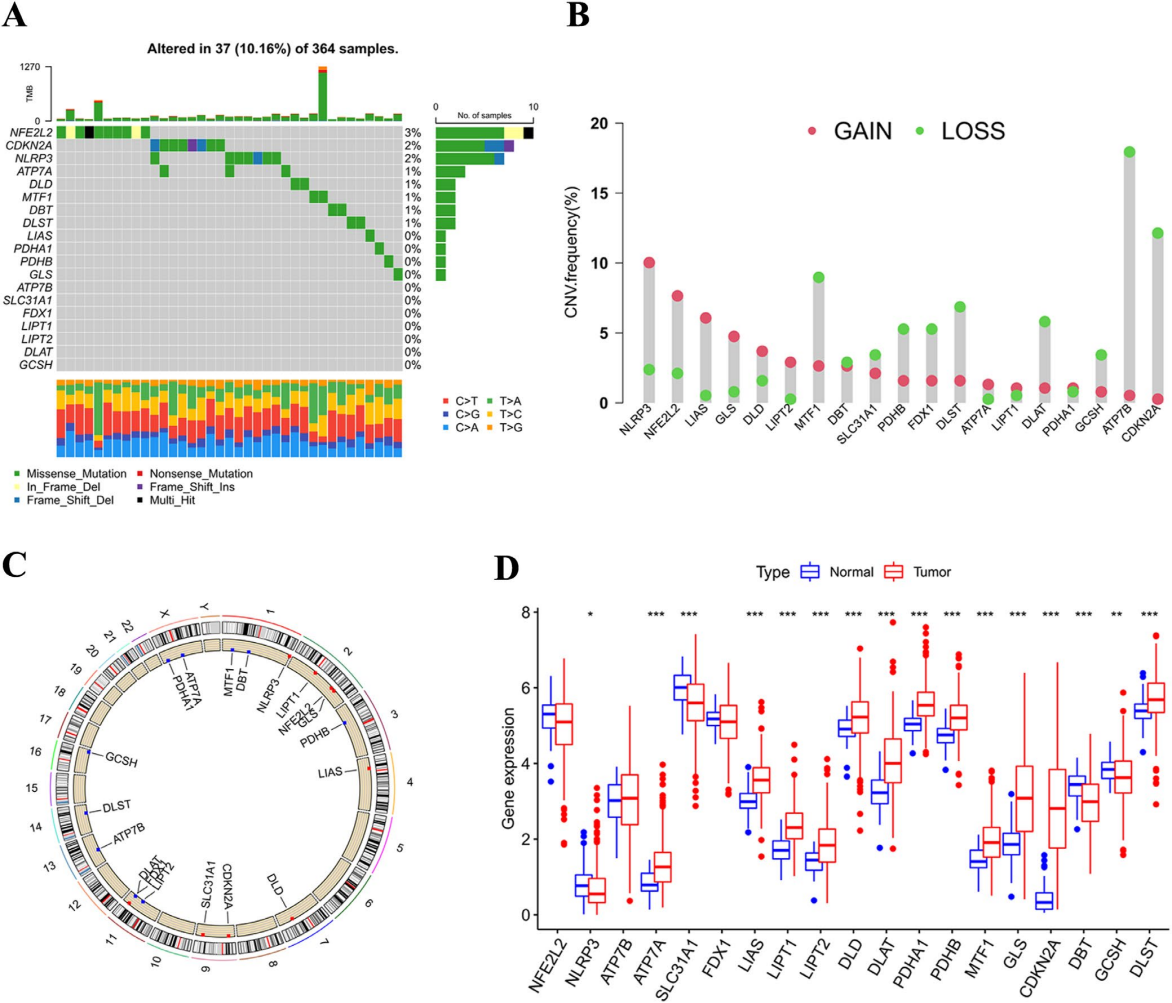
Genetic profile of cuproptosis‐related genes in HCC. (A) Mutation frequency spectrum of 19 CRGs in TCGA cohort, and the modules below correspond to different mutation types. (B) CNV frequency in the TCGA cohort for 19 CRGs. Red represents amplification and green represents deletion. (C) CRGs at sites of CNV change in 23 human chromosome pairs. (D) CRGs were expressed between HCC tissues and normal tissues. *p < 0.05, **p < 0.01, ***p < 0.001. CRGs, cuproptosis‐related genes; CNV, copy number variation; HCC, hepatocellular carcinoma; TCGA, The Cancer Genome Atlas.

As previously described, 19 genes are thought to be associated with cuproptosis [13]. To explore the role of these genes in HCC, we described the genetic profile of CRGs. Mutations in cuproptosis‐related loci occurred in 37 of 364 HCC patients (10.16%) (Figure 1A). The frequency of CNV amplification in nine genes was higher than that of deletion, and the frequency of CNV deletion in 10 genes was higher than that of amplification (Figure 1B,C). Comparing HCC to normal tissue, most CRGs show differences (Figure 1D). The expression of 15 CRGs was significantly different from the prognosis of HCC patients (Figure S1A–O). These specificities revealed that the different expression of CRGs was closely related to HCC progression.

### 3.2 Consensus Clustering Analysis Identified Three Cuproptosis Clusters

**FIGURE 2:**
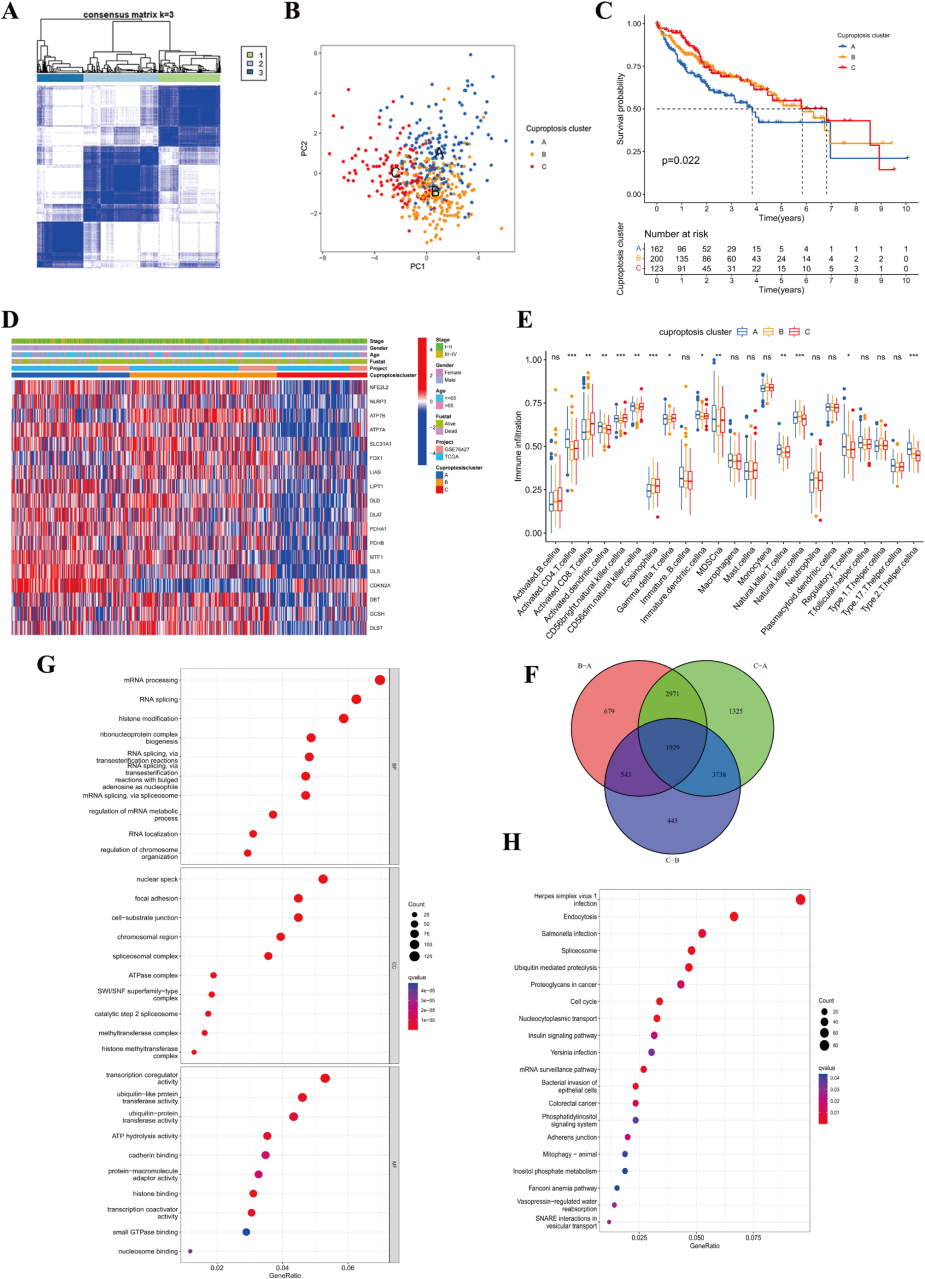
HCC subgroups associated with cuproptosis. (A) After consensus clustering analysis, all samples were divided into three cuproptosis clusters. (B) Relative distribution of the three cuproptosis clusters in PCA analysis. (C) Kaplan–Meier curves of the three cuproptosis clusters show the difference in survival (*p* = 0.022). (D) Expression of CRGs in three cuproptosis clusters. Red indicates high expression and blue indicates low expression. (E) Infiltrate abundance of ICI in the three cuproptosis clusters (***p < 0.001, **p < 0.01, *p < 0.05). (F) Differentially expressed DEGs in the three cuproptosis clusters. (G) and (H) GO and KEGG analysis of 1929 DEGs showed enriched pathways. Red represents activated pathways and blue represents inhibited pathways.

Based on the expression data of 19 CRGs, the consensus clustering algorithm is used to cluster the samples to obtain three cuproptosis clusters: A, B and C (Figure 2A). The PCA analysis results intuitively showed the obvious subpopulation differentiation among the three clusters (Figure 2B), which confirmed the reliability of our clustering [27]. The prognosis of cluster A was worse than that of B and C (Figure 2C, *p* = 0.022). The expression of CRGs in the samples of cluster A and cluster B was higher than that of cluster C, but there was no significant difference among the three clusters in terms of grade, age, sex, survival status and data set (Figure 2D). GSVA was applied to analyse the biological behaviour of three clusters. Cluster A was mainly enriched in cancer pathways, signal pathways, cell cycle and other pathways (Figure S2A), cluster B was not only enriched in cancer but also related to signal pathways and tumour apoptosis pathways (Figure S2C), while cluster C was mainly related to metabolic pathways (Figure S2B). The difference of immune cell infiltration (ICI) between groups is shown in Figure 2E. The results of the three control groups were combined to obtain 1929 coexpressed DEGs (Figure 2F). Go functional analysis showed that DEGs were mainly enriched in epigenetic modification pathways (Figure 2G). KEGG found that these genes were mainly related to cell cycle, protein ubiquitination, cell transport and other pathways (Figure 2H). These results suggest that cuproptosis plays an important role in tumour TME.

### 3.3 Clustering of Cuproptosis DEGs May Have Better Predictive Prognosis

**FIGURE 3:**
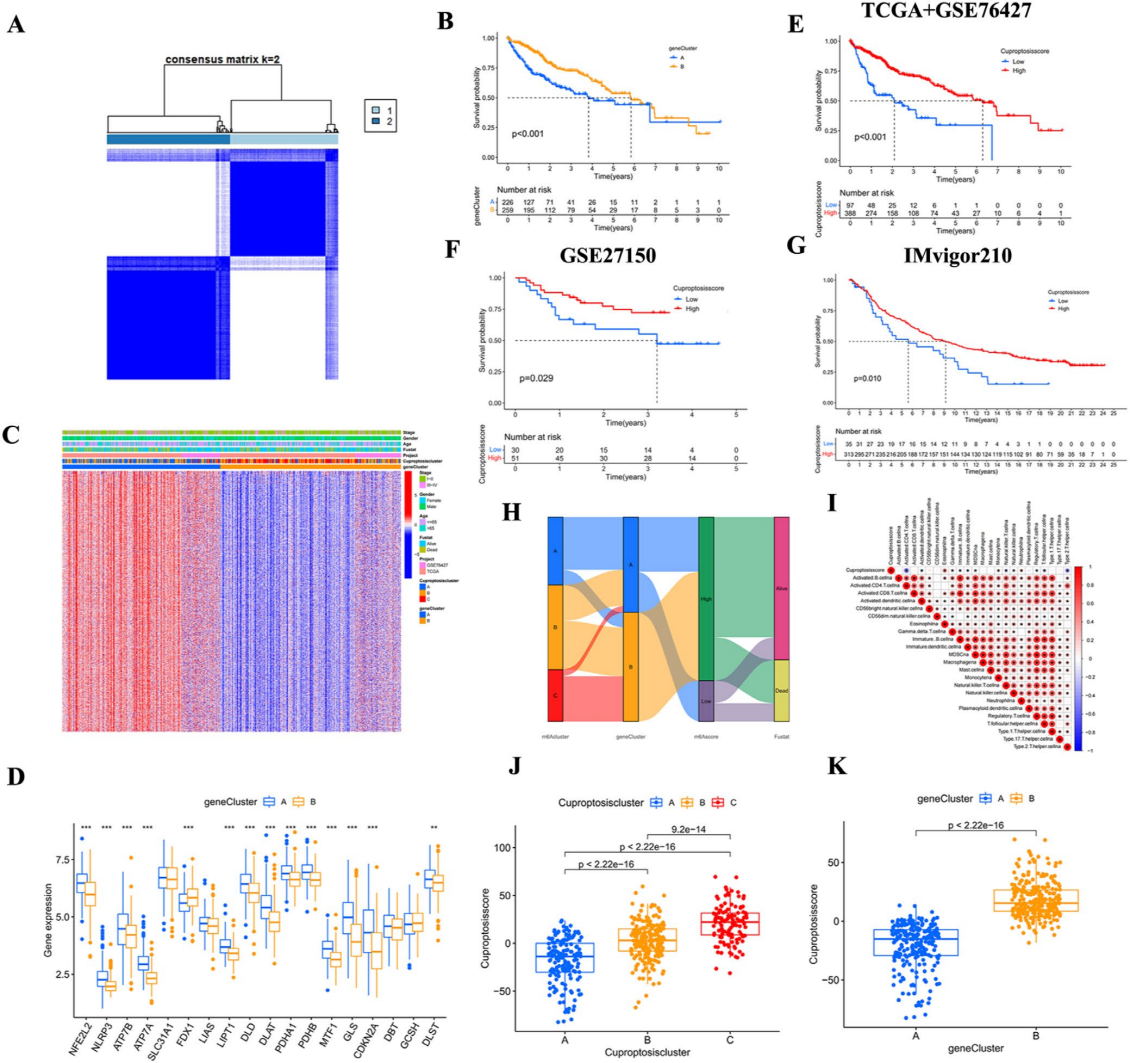
Clustering of cuproptosis DEGs may have better predictive prognosis. (A) After consensus clustering analysis, all samples were separated into two gene clusters. (B) Kaplan–Meier curves between the two gene clusters showed survival differences (*p* < 0.001). (C) Expression heatmap of the cuproptosis‐associated DEGs in different cuproptosis clusters and gene clusters. Red indicates high expression and blue indicates low expression. (D) Expression differences of CRGs in gene clusters (***p < 0.001, **p < 0.01). (E) Kaplan–Meier curves of HSG and LSG in TCGA and GSE76427 cohorts showed differences in overall survival (*p* < 0.001). (F) Kaplan–Meier curves for HSG and LSG in the GSE27150 cohort (*p* = 0.029), and (G) for the IMvigor210 cohort (*p* = 0.010) show differences in overall survival. (H) The distributions of the cuproptosis clusters, gene clusters, cuproptosis score and survival status are shown in the Sankey diagram. (I) Correlation between CS and each ICI infiltration. Red indicates positive correlation and blue indicates negative correlation. (J) Differences in the CS of the three cuproptosis clusters (*p* < 0.001). (K) Differences in the CS of the two gene clusters (*p* < 0.001).

Through univariate Cox analysis of DEGs, 1368 genes significantly associated with prognosis were screened out. Based on the expression of these prognostic genes, unsupervised cluster analysis was conducted again on the samples to obtain two clusters (Figure 3A). The survival prognosis of cluster B was better than that of cluster A (Figure 3B, *p* < 0.001). Most samples in cluster A belong to cuproptosis cluster A. The highly expressed gene pairs were concentrated in cluster A, while the expression in cluster B was significantly lower (Figure 3C). Similarly, the expression of CRGs in cluster A is higher than that in cluster B (Figure 3D).

### 3.4 The Construction of Cuproptosis Score Had Good Predictive Value for Prognosis

Based on the CS of 485 HCC samples obtained, the samples were divided into HSG group and LSG group according to the cut‐off value. Compared with LSG group, patients in HSG group had better prognosis (Figure 3E, *p* < 0.001). GSE27150 dataset and IMvigor210 cohort data are used to verify the accuracy of CS in predicting prognosis. The prognosis of HSG was better than LSG in the GSE27150 dataset (Figure 3F, *p* = 0.029), and in the IMvigor210 cohort also HSG had a better prognosis (Figure 3G, *p* < 0.001). The majority of patients with better prognosis in cuproptosis clusters B and C belong to gene cluster B. In contrast to gene cluster A, the majority of patients with better prognosis in gene cluster B belong to HSG, and the majority of patients with HSG are ALIVE (Figure 3H).

The relationship between CS and ICI is also depicted visually (Figure 3I). Among the different cuproptosis clusters, cluster C scored significantly higher than A and B (Figure 3J, *p* < 0.001). Among the different gene clusters, the score of cluster B was significantly higher than that of cluster A (Figure 3K, *p* < 0.001).

### 3.5 Cuproptosis Score was Correlated With TMB, MSI and Clinical Features

**FIGURE 4:**
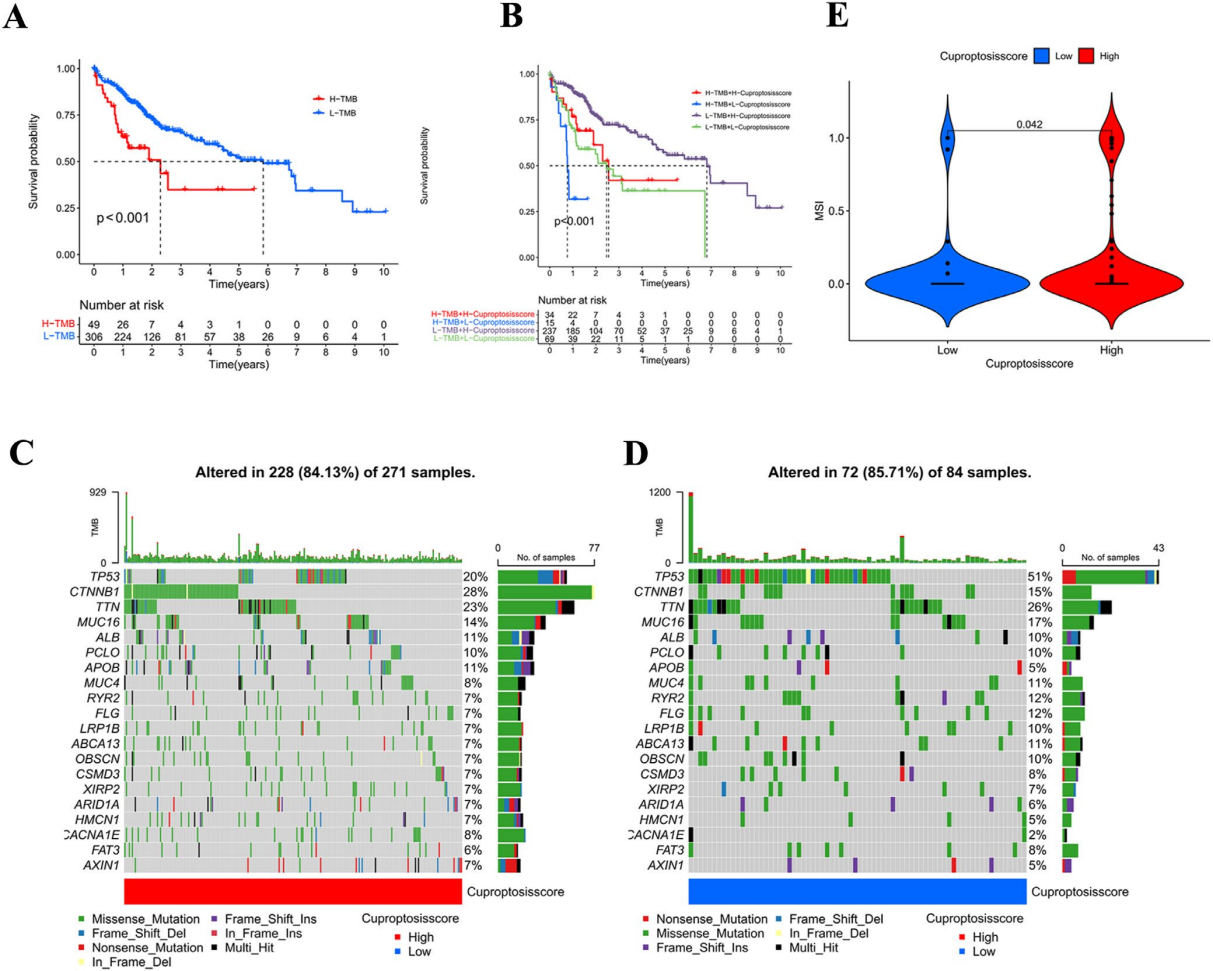
Cuproptosis score was correlated with TMB, MSI. (A) Kaplan–Meier curves of TMB subgroups show the difference in overall survival (*p* < 0.001). (B) Kaplan–Meier curves for the TMB and CS subgroups showed differences in overall survival (*p* < 0.001). (C) Top 20 alleles by mutation frequency in HSG and (D) LSG groups. The lower modules are for mutation type. (E) The difference between HSG and LSG in MSI score (*p* = 0.042).

Previous enrichment analyses noted that cuproptosis clustering was associated with genetically variant pathways. We investigated the association of CS with TMB and MSI. The expression of TMB did not differ in HSG versus LSG (Figure S3A,B), whereas in HCC samples, survival was better in the low mutation group (L–TMB) than in the high mutation group (H–TMB) (Figure 4A, *p* < 0.001). Samples with L–TMB and high cuproptosis score groups had the best survival, and samples with H–TMB and L–cuproptosis scores had the worst prognosis (Figure 4B, *p* < 0.001). From these results, we can conclude that low mutation and high cuproptosis scores play a synergistic role in prognostic evaluation.

We investigated the mutation spectrum in HSG and LSG samples and obtained maftools for the 20 genes with the top mutation frequencies in both groups. In the HSG group, 228 (84.13%) of 271 HCC samples were mutated, and the genes with higher mutations were frequently mutated in tumours, such as TP53, CTNNB1, TTN and MUC16 (Figure 4C). In the LSG group, 72 (85.71%) of 84 samples were variably mutated (Figure 4D). Analysis of the relationship between CS and MSI scores showed that MSI scores were higher in the HSG samples compared to the LSG group (Figure 4E, *p* = 0.042).

In addition, to explore the relationship between CS and clinical features more deeply, we analysed the differences in cuproptosis scores among traits. Patients with high scores had a significantly increased probability of surviving with lower scores (Figure S4A,B). Among samples with different stages, patients with advanced stages (III−IV) had significantly lower scores than those with earlier stages (I−II) (Figure S4C,D). The CS also has predictive power for patient staging. CS also shows differences in age and sex (Figure 4E–H). The higher CS score in patients with longer survival further illustrates the accuracy of the score. These results are a deeper impression of the clinical value of CS for tumour progression versus prediction.

### 3.6 Cuproptosis Score Had Considerable Potential in the Prediction of Tumour Treatment Effect

**FIGURE 5:**
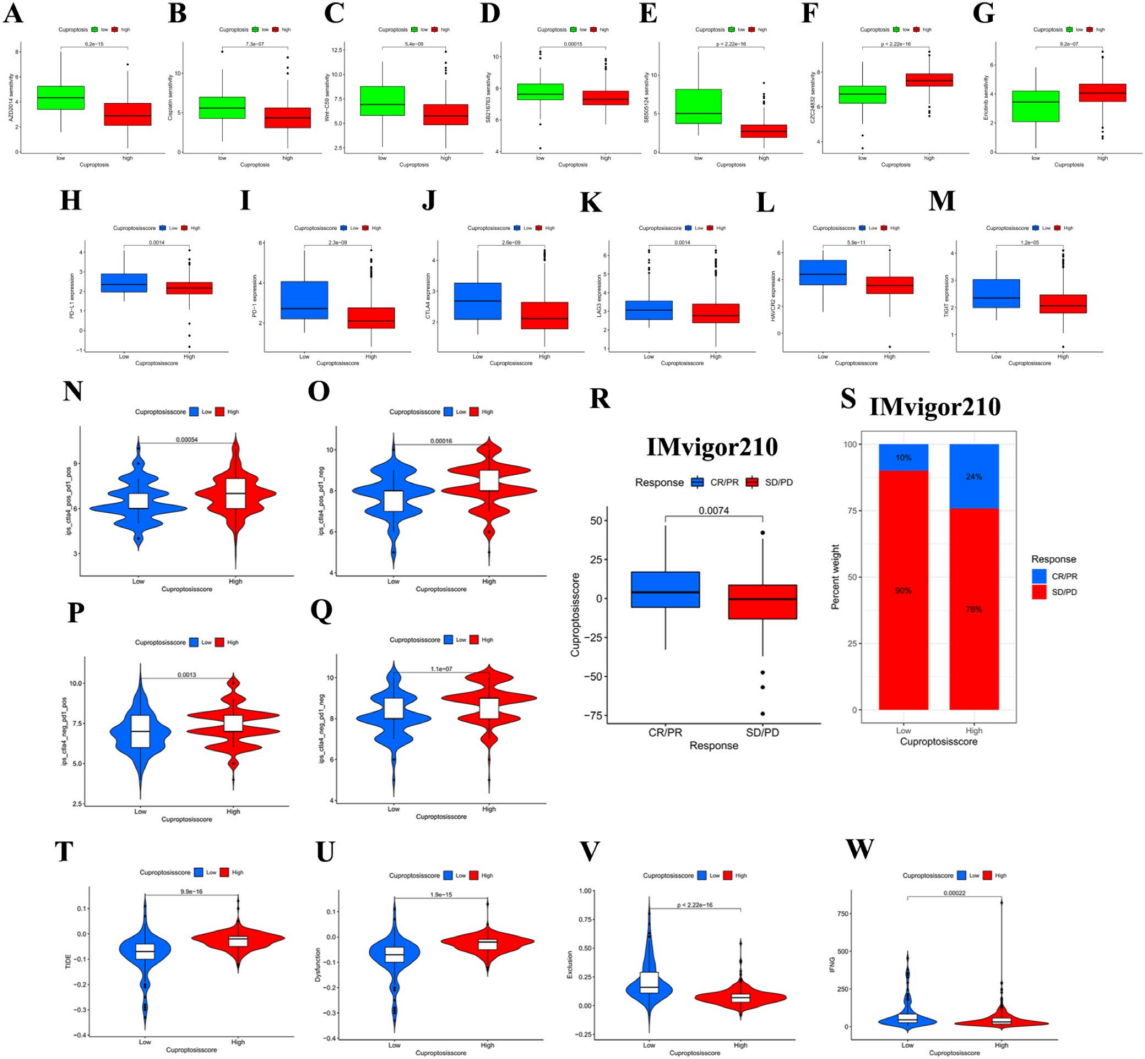
Cuproptosis score had considerable potential in the prediction of tumour treatment effect. (A–G) Sensitivity differences of multiple drugs in HSG and LSG. AED2014 (A), Cisplatin (B), Wnt‐C59 (C), SB216763 (D), SB505124 (E), CZC24832 (F), Erlotinib (G), *p* < 0.001. (H–M) Common immune checkpoint genes are differentially expressed in HSG and LSG. PD1 (H), PD‐L1 (I), CTLA4 (J), LAG3 (K), HAVCR2 (L), TIGIT (M), all *p* < 0.01. (N–Q) Differences in IPS between HSG and LSG. CTLA4 positive and PD1 positive (N), CTLA4 positive and PD1 negative (O), CTLA4 negative and PD1 positive (P), CTLA4 negative and PD1 negative (Q), all *p* < 0.01. (R) Difference in CS score for treatment response in the IMvigor210 cohort (*p* < 0.01). (S) Percentage of different treatment response groups in IMvigor210 cohort. (T–W) Differences of each immune score in HSG and LSG. TIDE. (T), Dysfunction. (U), Exclusion. (V), IFNG(W), *p* < 0.001.

Immunotherapy for HCC has been the focus of much attention. We analysed the association between CS subgroups and drug treatment efficacy. From the results of OncoPredict drug sensitivity analysis, among AED2014 (mTOR inhibitor), Cisplatin (cisplatin inhibitor), Wnt‐C59 (Wnt inhibitor), SB216763 (GSK‐3 inhibitor) and SB505124 (TGFβR inhibitor), the HSG group had significantly lower drug sensitivity scores than the LSG group (Figure 5A–E, *p* < 0.001) and higher sensitivity. In CZC24832 (P13K inhibitor) and Erlotinib (EGFR inhibitor), the drug sensitivity in the LSG group was significantly lower than that in the HSG group (Figure 5F,G, *p* < 0.001). It can be seen that different drugs have sensitivity differences in the two groups, and most of the drugs have higher sensitivity in the HSG group, which may be prominent for the treatment effect. The expression of immune checkpoint genes PD1, PD‐L1, CTLA4, LAG3, HAVCR2 and TIGIT in HSG was significantly lower than that in LSG group (Figure 5H–M, *p* < 0.01).

In the CTLA4‐positive and PD1‐positive, CTLA4‐negative and PD1‐negative, CTLA4‐negative and PD1‐positive and CTLA4‐negative and PD1‐negative groups, the IPS in HSG was significantly higher than that in LSG (Figure 5N–Q, *p* < 0.01). Patients in the HSG group responded better to CTLA4 and PD1, which predicted a good prognosis. Another immunotherapy evaluation showed that the CS score of CR/PR patients was significantly higher than that of SD/PD patients (Figure 5R,S).

TIDE score assesses the clinical response to immune checkpoint inhibitor therapy. A high TIDE score predicts a low immune checkpoint blocker (ICB) response and an unsatisfactory ICB oncologic outcome. The analysis showed that the TIDE score and dysfunction score in HSG group were higher than those in LSG group (Figure 5T,U, *p* < 0.001). In contrast, the Exclusion score and IFNG score in HSG group were significantly lower than those in LSG group (Figure 5V,W, *p* < 0.001). The TIDE score comprises two components: Dysfunction score and exclusion score. The dysfunction score evaluates whether there are functional impairments in T cells within the tumour microenvironment. The immune exclusion score assesses whether immune cells, particularly T cells, are excluded from the tumour microenvironment, preventing them from infiltrating the tumour and mounting an attack. The TIDE score combines these two aspects to provide an overall prediction of a tumour's response to immune checkpoint inhibitors. A higher TIDE score typically signifies a poorer treatment response and prognosis. Therefore, despite the lower immune rejection score in the HSG group, the higher TIDE score indicates a worse response to ICI treatment. In other words, although immune exclusion may be weaker in the HSG group, the dysfunction of immune cells leads to a poorer response to ICI treatment. Therefore, in the clinical treatment of HCC, we should be more cautious about the use of ICI treatment.

### 3.7 GLS Identified as a Cuproptosis‐Related Prognostic Key Gene by WGCNA Analysis

**FIGURE 6:**
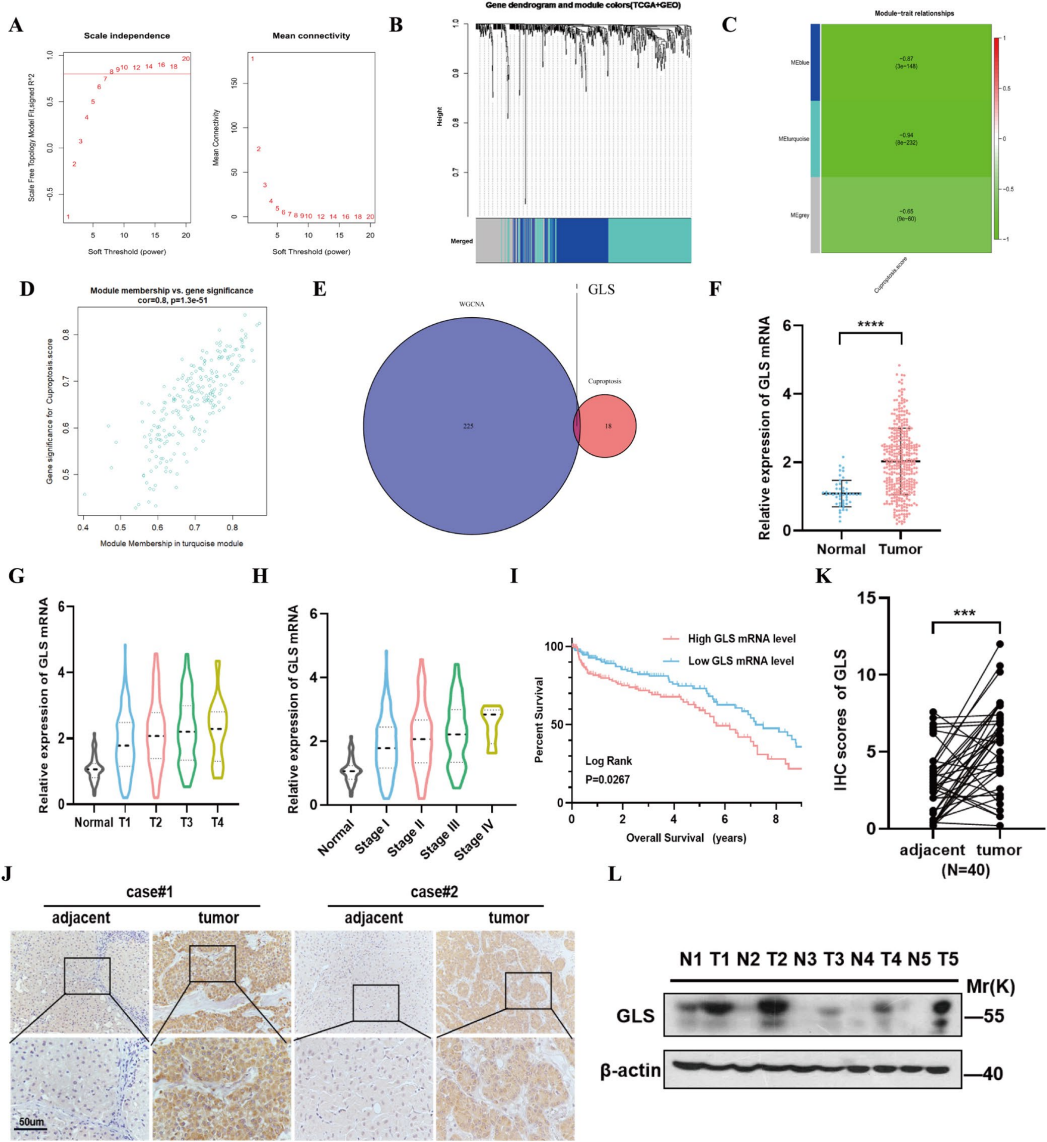
GLS identified as a cuproptosis‐related prognostic key gene by WGCNA analysis. (A) The optimal power value of 10 was selected to achieve coexpression analysis. (B) The branches of the dendrogram correspond to three significant gene modules. (C) Correlation between significant gene modules and CS. (D) Scatter plot of module eigengenes in the turquoise module. (E) The turquoise module genes were intersected with CRGs to obtain the key genes. (F) The expression of GLS in HCC tissues versus normal tissues differed at the mRNA level (*p* < 0.001). (G, H) The expression of GLS was correlated with clinical features. (I) Kaplan–Meier curves showed differences in overall survival between high and low expression of GLS protein levels (*p* = 0.027). (J, K) GLS staining was stronger in HCC tissues (*n* = 40) than in paracarcinoma tissues (*n* = 40). Typical IHC images were displayed here. (L) Western blotting showed the difference in GLS protein level between HCC and adjacent tissues.

To identify prognostic key genes under the cuproptosis scoring pattern, WGCNA was used to analyse 1929 coexpressed genes. The most obvious module with CS traits was the turquoise module (Figure 6A–D, correlation coefficient = −0.94, *p* < 0.001). We crossed the turquoise module with CRGs to obtain the key gene GLS (Figure 6E). RNA data from TCGA suggested that the expression of GLS in HCC was significantly increased (Figure 6F, *p* < 0.001) and was correlated with clinical features (Figure 6G,H). Furthermore, individuals with higher GLS expression had a considerably shorter overall survival than patients with lower levels (Figure 6I, *p* = 0.027). We analysed the expression of GLS in human HCC and adjacent normal tissues from our centre. Immunohistochemistry (IHC) in 40 paired tissues from our centre displayed that GLS expression was higher in HCC tissues than the adjacent normal tissues. Two pairs of typical IHC images are displayed here (Figure 6J,K).

The verification results of five pairs of HCC tissue and adjacent liver tissue samples also illustrate this conclusion (Figure 6L). These results indicate that GLS is a key prognostic gene associated with copper poisoning.

### 3.8 GLS Expression Enhances HCC Cell Proliferation, Motility and EMT Progression

**FIGURE 7:**
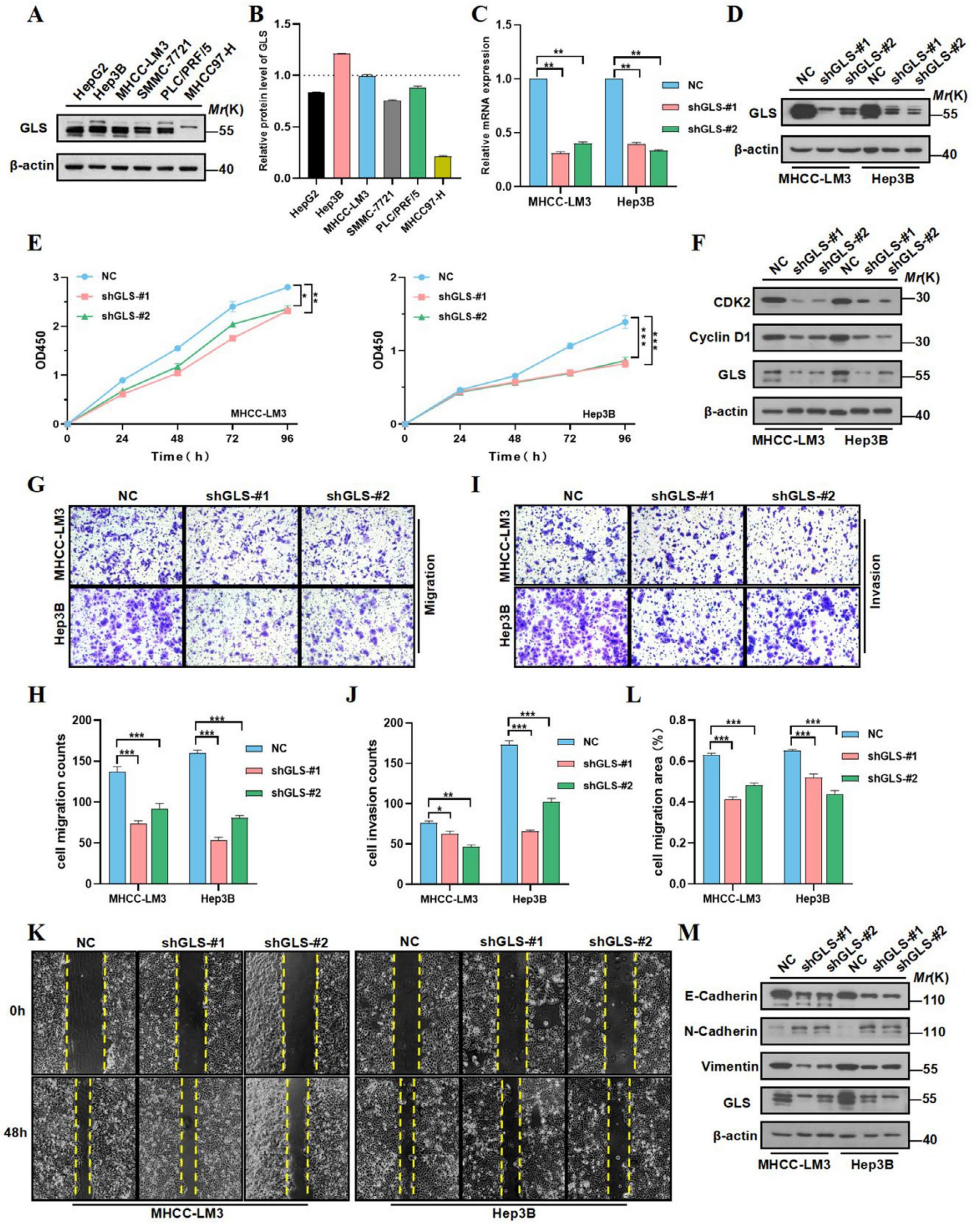
GLS expression enhances HCC cell proliferation, motility and EMT progression. (A, B) The protein expression level of GLS in HCC cell lines was detected by western blotting. (C, D) The knockdown of GLS was validated by western blotting and qRT‐PCR. (E) CCK8 assay was used to verify GLS knockdown can inhibit the proliferation ability. (F) Following GLS deletion, Cyclin D1 and CDK2 expression levels were markedly lower. (G–K) The migration and invasion ability of HCC cells were compared by wound healing assay and transwell assay after GLS knockout. (L, M) Measure the expression of various proteins closely associated with EMT in GLS knockdown group.

To evaluate the role of GLS in the malignant biological behaviour of HCC. We looked at GLS expression levels in six distinct HCC cell lines. It was discovered that it was greater in LM3 and Hep3B cell lines (Figure 7A,B). Then, we use shRNA to deplete the endogenous expression of GLS in LM3 and Hep3B. Western blotting and qRT‐PCR assays were used to prove that the knockdown cell lines were successfully constructed (Figure 7C,D). CCK‐8 experiment showed that compared with the control group, GLS depletion dramatically slowed down the proliferation of LM3 and Hep3B cells (Figure 7E). In addition, we measured the expression of some proteins closely related to cell cycle, and found that following GLS deletion, cyclin D1 and CDK2 expression levels were markedly lower (Figure 7F). Therefore, we believed that downregulation of GLS could inhibit the malignant proliferation of HCC cells.

To illustrate the function of GLS in promoting HCC cell motility, we investigated the effect of GLS on migration and invasion of LM3 and Hep3B cells by transwell assays and wound healing. The GLS knockdown group had slower scratch healing ability and poorer migration and invasion ability (Figure 7G–K). Then, we examined the expression of some indicators of epithelial–mesenchymal transition (EMT) process (E‐cadherin, N‐cadherin, vimentin), which are so closely related to tumour metastasis. We further found that the expression of N‐cadherin and vimentin was significantly reduced in the GLS gene downregulation group, while the expression of E‐cadherin was significantly increased (Figure 7M), indicating that GLS could promote the EMT process of CRC cells.

## 4 Discussion

As a common malignancy, HCC is usually asymptomatic and rapidly progressive, and patient prognosis is generally poor. Patients with early‐stage HCC are usually treated with traditional therapies: surgical resection, radiofrequency ablation, TACE [28, 29, 30]. The most effective radical treatment for HCC is recognised as liver transplantation, which is not widely available due to the lack of liver resources. Only palliative treatments are available for patients with advanced disease. With the rise of immunotherapy in recent years, treatment options for patients with advanced HCC have progressed significantly. According to many studies, the prognosis and treatment responsiveness of HCC patients are closely related to the immune cell component [31, 32, 33, 34, 35, 36].

Our CS is of great value for the evaluation and prediction of tumour treatment outcomes. Patients in the HSG group were more sensitive to AZD2014, cisplatin, Wnt‐C59, SB216763 and may have better treatment outcomes. Patients in the LSG group were more sensitive to CZC24832 and Erlotinib AZD2014 has shown antitumour effects as an mTOR inhibitor in a variety of cancers including prostate cancer and HCC [37, 38]. Tumour proliferation, EMT process and apoptosis of tumour cells in a nude mouse model of HCC in vivo are the result of AZD2014 inhibition [39]. AZD2014 also exerts mTOR inhibition and arrests the cell cycle to increase sensitivity of oral squamous cell carcinoma to radiotherapy [40]. Cisplatin is a familiar anticancer drug used in various solid tumours and is now widely used in lung, ovarian, breast, kidney and head and neck cancers [41]. The Wnt pathway inhibitor Wnt‐C59 inhibits growth via TME in mouse models of nasopharyngeal carcinoma [42]. The predicted sensitivity of these drugs in HSG and LSG will help us to target patients for precise treatment.

Studies targeting immune checkpoints are an important direction in current tumour therapy. Higher expression of common immune checkpoints in the LSG group may provide better benefit from immune checkpoint inhibitor therapy. Immunophenotype scores in the HSG group are higher in CTLA4 and PD1 drugs and have better therapeutic response. Subtypes in melanoma against PD1 survival benefit of anti‐CTLA4 therapy in studies suggesting biological subtypes used as independent predictive markers to aid clinical decision making [43]. Patients who reached the CR or PR stage had higher CS scores, while those who only reached SD or PD had lower scores. Patients with HSG tumours recovered significantly better, and the scores can be used to assess the efficacy of oncologic therapy for patients. The results of the TIDE score calculation confirm that patients with HSG have a lower and more effective rejection response to ICI therapy. The immune dysfunction score and rejection score can be used to clinically guide the use of medications and improve the accuracy of patient treatment.

Because of the important role of copper toxicity typing patterns in tumour prognosis and immunotherapeutic response, we identified the prognostic key gene GLS. GLS, a key enzyme of glutamine metabolism, is overexpressed in several tumour cells, and targeting GLS significantly inhibits tumour cell proliferation [44]. Tumour cells require energy materials from their environment for growth and development, and glutamine metabolism provides a carbon source for TCA in tumour cells, and research has received attention in recent years targeting blocking its metabolism [45]. CB‐839 (Telaglenastat), an inhibitor of glutaminase, has been the subject of numerous oncology clinical drug trials. Proliferation of prostate cancer can be attenuated by inhibition of GLS, which acts as an oncogene in PCa and could be a future therapeutic target [46]. It is evident that GLS acts as a tumour‐promoting factor in tumours. It was first proposed that GLS is a cuproptosis‐related gene in 2022. To identify the specific metabolic pathways that mediate copper toxicity, this research performed genome‐wide CRISPR‐Cas9 loss‐of‐function screens to identify the genes involved in copper ionophore–induced death. The results clearly indicate that GLS is a negative regulator of cuproptosis [13]. Furthermore, according to Ning et al., they developed a nanosystem that can effectively suppresses GLS activity, thereby reducing GSH content, enhancing cuproptosis and immune modulation [47]. GLS in our study also showed similar results: GLS with high expression in HCC had a poor prognosis. These suggest that cuproptosis‐associated key gene GLS is a new biological marker for HCC diagnosis and prognosis.

However, the current study still has some limitations; firstly, our study needs more independent cohorts with larger sample sizes to validate the accuracy of CS, although some independent cohorts have been used for validation in this study. Secondly, the mechanism and role of the key gene GLS in HCC need further experiments to be discovered.

In general, our study is the first to combine the association between cuproptosis typing patterns in HCC and ICI. Cuproptosis typing identification may help to understand the immune infiltration in patients who do not use it. For this purpose, we quantitatively constructed the cuproptosis score, CS has accurate prediction in prognosis assessment, drug sensitivity, immunotherapy response in tumour patients and is a new biological marker for prognosis and treatment. This study also identified GLS as a key prognostic gene related to cuproptosis, and high expression of GLS prognostic check, which may provide new targets and directions for future prognosis and treatment of HCC. In conclusion, this study provides a novel quantitative cuproptosis pattern score, the same for predicting prognosis and immunotherapy prediction, to facilitate clinicians to develop precise and individualised treatment plans.

## Author Contributions

**Kai Guo:** conceptualization (equal), funding acquisition (equal), methodology (equal), validation (equal), visualization (equal), writing – original draft (equal). **Tianbing Wang:** conceptualization (equal), methodology (equal), validation (equal), visualization (equal), writing – original draft (equal). **Jimin Yin:** conceptualization (equal), formal analysis (equal), methodology (equal), visualization (equal), writing – original draft (equal). **Shoushan Yang:** investigation (equal), methodology (equal), resources (equal), validation (equal), visualization (equal). **Haodong Cui:** data curation (equal), formal analysis (equal), resources (equal), validation (equal). **Zichuan Cao:** data curation (equal), investigation (equal), resources (equal), visualization (equal). **Qiang Zhao:** data curation (equal), investigation (equal), resources (equal). **Gongbo Xie:** data curation (equal), investigation (equal), resources (equal). **Jian Lu:** data curation (equal), investigation (equal), resources (equal). **Guosheng Gu:** methodology (equal), project administration (equal), supervision (equal), writing – review and editing (equal). **Wenyong Wu:** conceptualization (equal), funding acquisition (equal), methodology (equal), project administration (equal), supervision (equal), writing – review and editing (equal).

## Ethics Statement

The Institutional Reviewer Board has approved the research protocol: The Biomedical Ethics Committee of Anhui Medical University (March 1, 2024, ID: 83240098) authorised the research plan, which complies with the Helsinki Declaration.

## Consent

Informed consent: Informed consent was obtained from tumour donors. Consent to Participate: Informed consent was obtained from all individual participants included in the study. Consent for publication: After reviewing the manuscript, all authors agreed to its publication in the current form.

## Conflicts of Interest

The authors declare no conflicts of interest.

## Supporting information


Figures S1–S4.

## Data Availability

Publicly available datasets were used in this study. This data can be found here: TCGA database (http://portal.gdc.cancer.gov/repository), GEO database (GSE76427 and GSE27150) (http://www.ncbi.nlm.nih.gov/geo/), UCSC Xena database (http://xena.ucsc.edu/), IMvigor210 cohort (http://research‐pub.gene.com/IMvigor210CoreBiologies/), TCIA database (https://tcia.at/), GDSC database (https://www.cancerrxgene.org/), TIDE database (http://tide.dfci.harvard.edu) and CPTAC database (https://proteomics.cancer.gov/programs/cptac).
