# Supplementary material for: Identification of Cuproptosis‐Related Patterns Predict Prognosis and Immunotherapy Response in Hepatocellular Carcinoma
Source: J Cell Mol Med. 2024 Dec 11;28(23):e70224. doi: 10.1111/jcmm.70224 (PMC11634814; doi:10.1111/jcmm.70224)
Supplement: Supplementary file 1 — Figures S1–S4. [file JCMM-28-e70224-s001.docx]

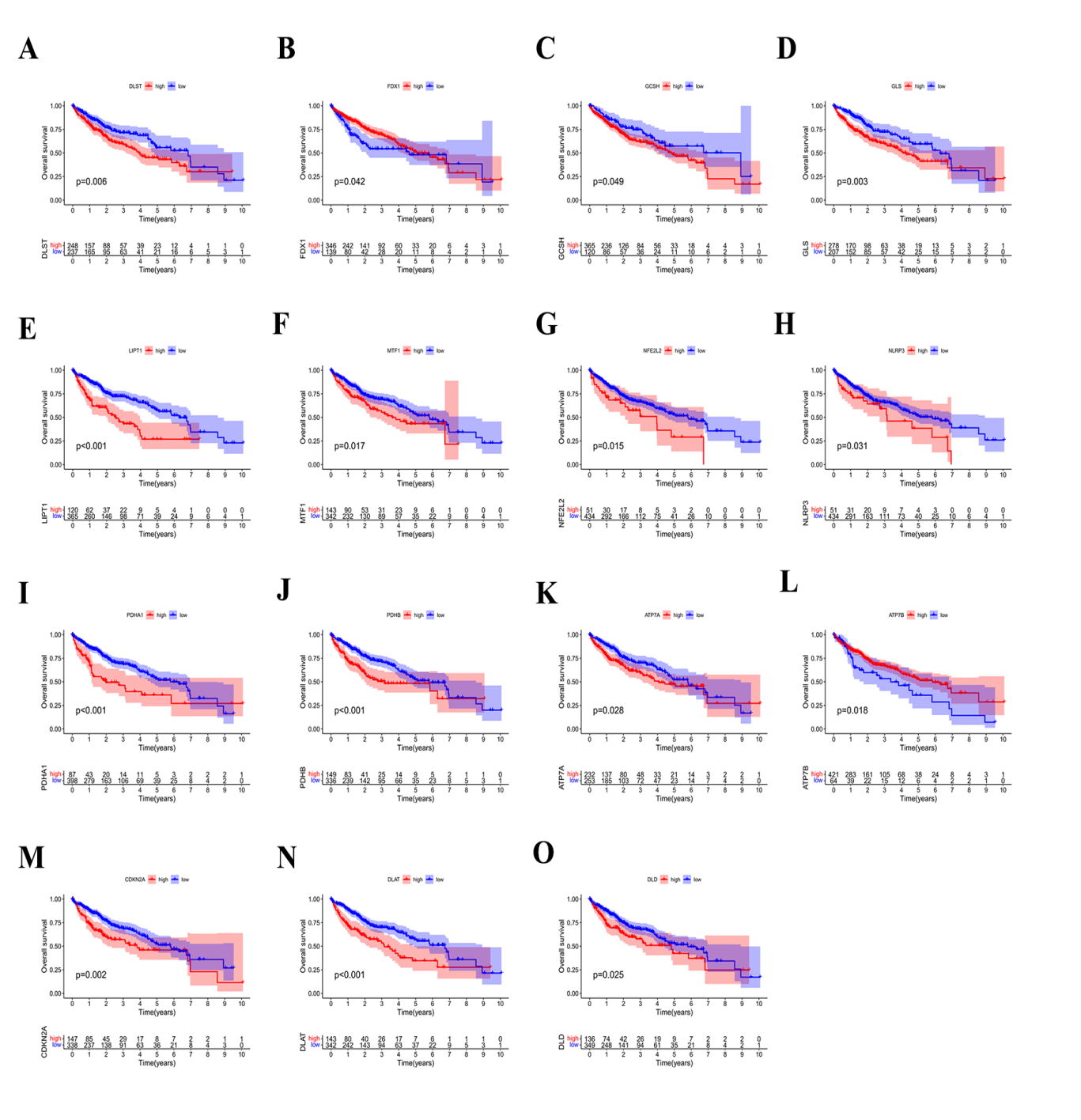


**Supplementary Figure 1** (A-O) High and low expression of 15 CRGs were significantly associated with poor overall survival. CRGs, cuproptosis-related genes.


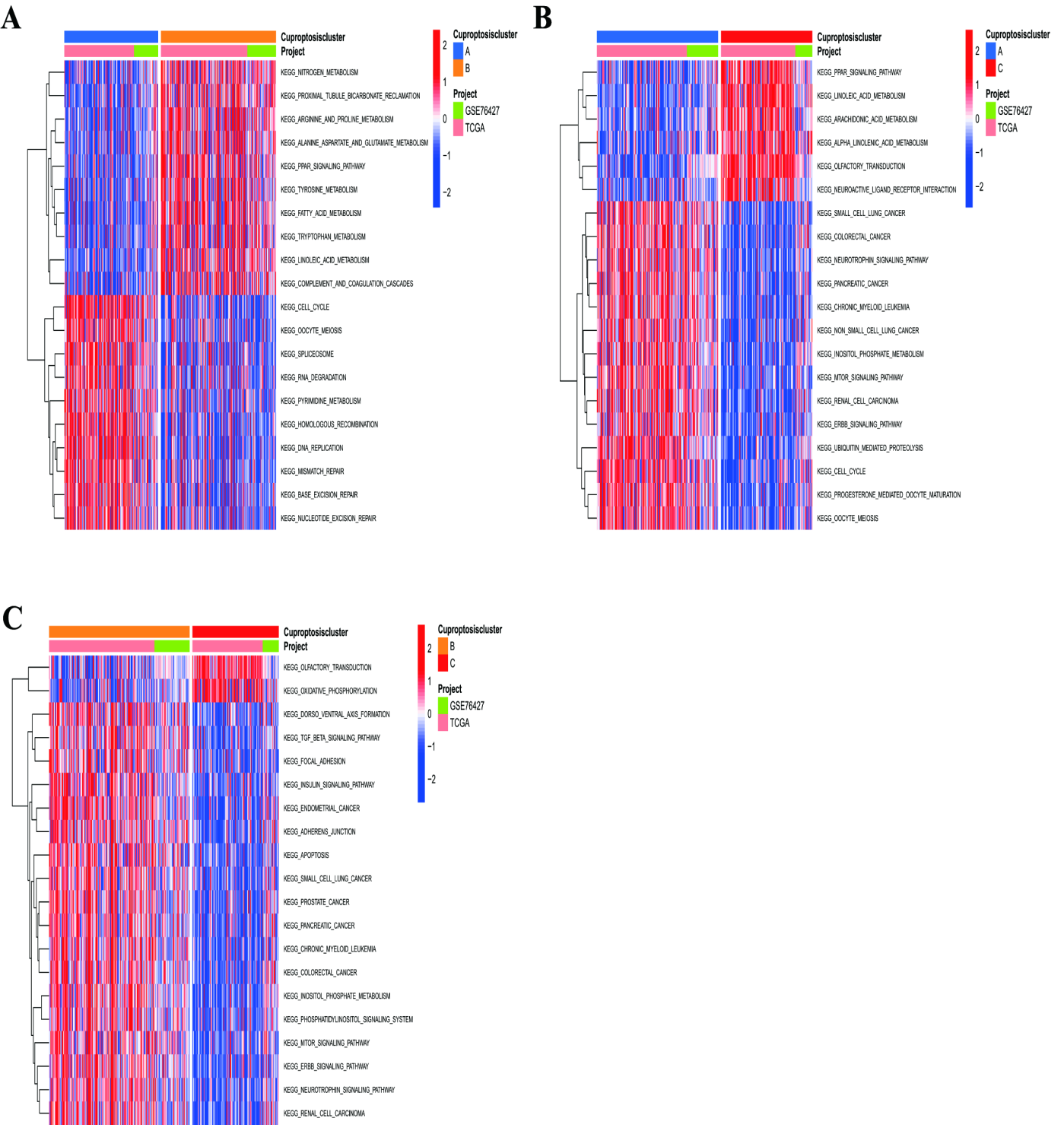


**Supplementary Figure 2** (A) GSVA shows pathways enriched for cuproptosis cluster A and cuproptosis cluster B. (B) GSVA shows pathways enriched for cuproptosis cluster A and cuproptosis cluste C. (C) GSVA shows pathways enriched for cuproptosis cluster B and cuproptosis cluste C.


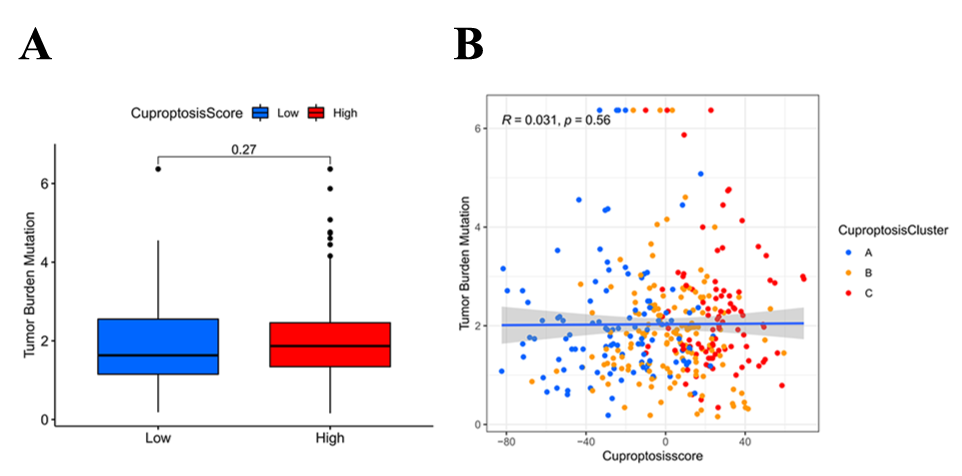


**Supplementary Figure 3** (A) There was no difference in TMB between HSG and LSG groups (p=0.27). (B) Correlation between CS and TMB across the three cuproptosis clusters (R=0.031, p=0.56).


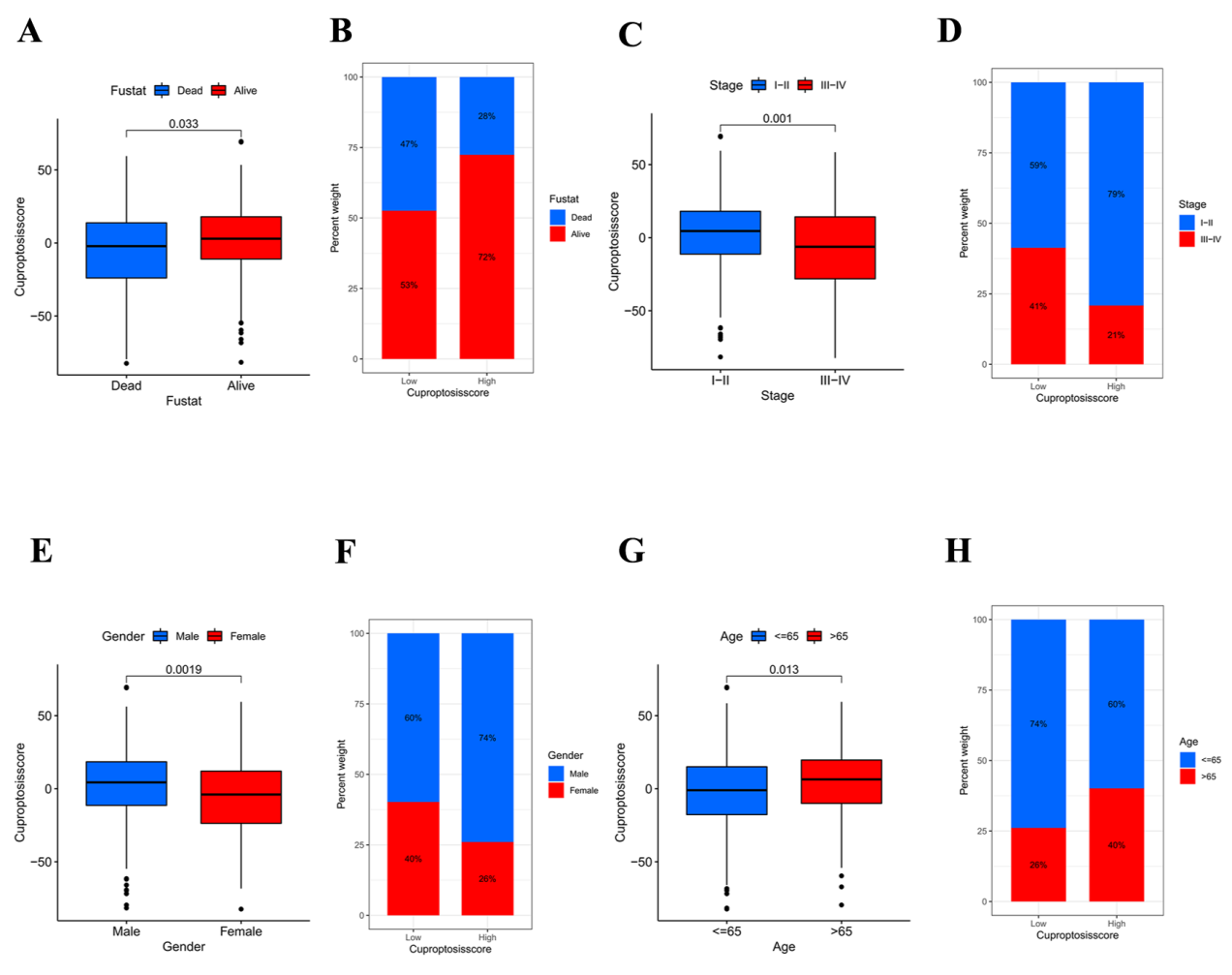


**Supplementary Figure 4** (A) CS score difference by survival status (p=0.033). (B) Percentage distribution of survival status in HSG and LSG samples. (C) The CS score differed between stages (p=0.001). (D) Percentage distribution of different stages in HSG and LSG samples. (E) The CS score differed between genders (p=0.0019). (F) Percentage distribution of different genders in HSG and LSG samples.(G) The CS score differed by age (p=0.013). (H) Percentage distribution at different ages in the HSG and LSG samples.
